# Priorities for End-of-Life Care Reporting in Nursing Homes: Results From a Mixed-Methods Study

**DOI:** 10.1093/geroni/igaa057.792

**Published:** 2020-12-16

**Authors:** Andrea Gruneir, Matthias Hoben, Charlotte Jensen, Monica Buencamino, Adam Easterbrook, Sheila Marshall, Janice Keefe, Carole Estabrooks

**Affiliations:** 1 University of Alberta, Edmonton, Alberta, Canada; 2 Staff, Edmonton, Alberta, Canada; 3 University of Alberta, Calgary, Alberta, Canada; 4 Centre for Health Evaluation and Outcomes Sciences, Vancouver, British Columbia, Canada; 5 University of British Columbia, Vancouver, British Columbia, Canada; 6 Mount Saint Vincent University, Halifax, Alberta, Canada

## Abstract

The aim of the “Trajectories” project is to compile measures of nursing home (NH) quality to better characterize the final year of life for residents. In the first phase, we worked with various stakeholder groups to identify their priorities to focus the selection of possible outcomes relevant to end-of-life needs. Policy- and decision-makers from 5 Canadian health regions participated in an on-line, modified Delphi process to reach consensus on 3-4 measures of each burdensome symptoms and potentially inappropriate care practices. NH residents and families or care aides participated in an interview process using the Action Project Method. To date, all participants identified pain, mental health care, polypharmacy, and dyspnea as priorities. Policy- and decision-makers additionally identified infections and acute care transfers as priorities, while residents and families additionally identified mobility, cognition, and pressure ulcer care as priorities. There was general consistency across groups in terms of priorities but additional measures seemed to reflect either a system-wide or more personal perspective, depending on the source. Data collection with frontline staff and managers is on-going. Moving forward, we will use this list of prioritized outcomes to quantitatively assess the trajectories of these outcomes and associated factors, and to create a profile that allows for monitoring of end-of-life care in NHs.

